# Comparison of three instruments used in the assessment of dementia in Sri Lanka

**DOI:** 10.4103/0019-5545.55957

**Published:** 2005

**Authors:** S.T. Kathriarachchi, S. Sivayogan, S.D. Jayaratna, S.R. Dharmasena

**Affiliations:** * Associate Professor, Department of Medicine, Faculty of Medical Sciences, University of Sri Jayewardenepura; ** Professor, Department of Medicine, Faculty of Medical Sciences, University of Sri Jayewardenepura; *** Senior Lecturer, Department of Medicine, Faculty of Medical Sciences, University of Sri Jayewardenepura; **** Demonstrator, Department of Medicine, Faculty of Medical Sciences, University of Sri Jayewardenepura

## Abstract

**Background:**

Dementia is poorly recognized even by physicians. This study compares three instruments used to assess dementia in a community setting in Sri Lanka.

**Method:**

Translated and culturally adapted versions of the Mini Mental State Examination (MMSE), Informant Questionnaire on Cognitive Decline in the Elderly (IQCODE) and Clinical Dementia Rating (CDR) were administered to 363 individuals selected by stratified random sampling in a periurban community in Sri Lanka. The results on the three instruments were compared with the independent psychiatric evaluation done on a concentrated sample of 37 individuals from the study population.

**Results:**

Culturally adapted MMSE, IQCODE and CDR can be used to screen dementia in Sri Lanka. IQCODE is the best among the three instruments with a sensitivity of 71.4% and a specificity of 82.6% when 3.5 is the cut-off. In addition, IQCODE is culturally acceptable, easy to administer and can be used in those with a low level of literacy as well as in those with hearing or visual impairment.

**Conclusion:**

IQCODE was found to be more effective and culturally acceptable as a screening tool for dementia in Sri Lanka, compared with MMSE and CDR.

## INTRODUCTION

The problem of dementia and the need for its early recognition has gained attention lately with new research focusing on various aspects of the problem.^1^ Physicians are able to diagnose only 50% of dementia cases.^2^ The identified barriers to the diagnosis of dementia in primary care include failure to recognize the symptoms, limited time and the perceived lack of need to determine a specific diagnosis.^2^ Assessment and recognition of dementia at the primary care level will ensure proper care to enhance the quality of life of both patients and caregivers. The use of screening instruments in an attempt to facilitate and improve recognition of dementia in primary care has shown promising results.^3^,^4^ In developing countries, a low level of education—both literacy and numeracy—can result in cognitively unimpaired people screening positive for dementia.^5^–^7^

In developed countries, different assessment tools have been used with a wide range of sensitivities and specificities. The most suitable instrument for each country will vary according to the different cultural and educational back-grounds. The Mini Mental State Examination (MMSE) is a widely used instrument, especially in screening for dementia.^8^ It is quick and easy to use. It cannot be used in those with hearing/visual impairment and in illiterate individuals. The Informant Questionnaire on Cognitive Decline in the Elderly (IQCODE) is designed to measure cognitive impairment depending on informants’ reports. It can be used in all patients irrespective of their physical condition or level of education.^9^ Clinical Dementia Rating (CDR) is a rating scale and can be used for serial measures of cognitive impairment.^10^

Of these, only the MMSE has been used in studies conducted in Sri Lanka. A culturally adapted Sinhalese translation of the MMSE used by De Silva *et al.* was found to be a sensitive screening tool for dementia in Sri Lanka.^11^

This paper compares the use of MMSE, IQCODE and CDR in the assessment of patients with dementia in Sri Lanka.

## METHODS

### Assessment tools

The MMSE consists of 11 questions with a maximum score of 30 points, with different domains assessed: orientation in time and place (10 points), registration of three words (3 points), attention and calculation (5 points), recall of three words (3 points), language (8 points) and visual construction (1 point). A score of <23 was taken as the standard cut-off point for dementia. This tool was used in a study conducted in Kerala, India.^12^ The culturally adapted Sinhalese translation was validated by De Silva *et al.* using a cut-off point of <17.^11^ For the present study, independent translation and validation of the MMSE was done using a nominal group technique^13^ and <23 was taken as the cut-off.

IQCODE is a questionnaire administered to an informant about changes in the cognitive function of an elderly person in day-to-day activities. It comprises 26 questions aiming to assess general cognitive decline independent of previous ability. One study used a standard cut-off score of >4 to screen patients with dementia.^9^

CDR is a global measure of dementia where 6 domains are assessed: memory, orientation, judgement and problem-solving, community affairs, home and hobbies, and personal care. CDR ratings are 0 for healthy people, 0.5 for questionable dementia and 1, 2 and 3 for mild, moderate and severe dementia, respectively.^10^

The MMSE, IQCODE and CDR were translated into Sinhalese, and re-translated into English by an independent person and matched for consistency. Minor changes in the questionnaires were made to make the questions culturally acceptable by a team comprising 2 psychiatrists, 1 community physician, 1 psychologist and 1 lecturer in social work. The questions were validated using a nominal group technique.^13^

### Study design

A semi-urban community from the areas of Raththanapitiya and Boralesgamuwa in the Colombo district was chosen for the study. It was selected as it was already mapped, was in close proximity allowing easy access and was not different from any other periurban community in the country. A serially numbered database that included all the households in this area (maintained at the Department of Community Medicine of the University of Sri Jayewardenepura) served as the sample frame. This database included details of all demographic factors of the individuals in the households including the age structure. All households with individuals >65 years of age were selected from this database and a stratified, computer-generated random sample of 400 households was selected as the study population.

The survey identified 400 people >65 years of age of which 363 (90.8%) were available for detailed evaluation. The rest were difficult to assess due to unavailability of the selected individuals and/or their carers, despite repeated attempts.

Clearance was taken from the Ethics Review Committee of the Faculty of Medical Sciences, University of Sri Jayewardenepura and informed consent obtained from all the participants.

The study was conducted in two phases. During phase I, 400 subjects ≥65 years of age in the study sample were traced by a door-to-door survey. A medical doctor trained by a consultant psychiatrist administered the study-specific questionnaire along with the three instruments to identify subjects with cognitive impairment. Those who were severely ill, unable to communicate or had visual/hearing disabilities were excluded.

In phase II, a sample of 40 individuals was selected for evaluation by psychiatrists. This was a concentrated sample of individuals who had scores ranging from normal to severe dementia on the rating scales. Two consultant psychiatrists evaluated these patients blindly and independently to identify the most suitable instrument to be used in the Sri Lankan setting.

The data were tabulated and analysed using the SPSS version 10.0 computer package.

## RESULTS

Of the 40 patients selected for evaluation by psychiatrists, 3 were not available for assessment due to death and change of place of stay. From the 37 patients evaluated, 14 were diagnosed clinically as definitely having dementia. Independent evaluation by the 2 psychiatrists correlated perfectly with no discrepancy in their diagnosis.

The sensitivity, specificity, and positive and negative predictive values were calculated using clinical assessment by the psychiatrists as the ‘gold standard’. Table 1 depicts these values.

**Table 1:** Comparison of the three instruments using clinical assessment as the ‘gold standard’

Instrument	Cut-off point	Sensitivity (%)	Specificity (%)	Positive predictive value	Negative predictive value	Likelihood ratio
IQCODE	>3	100.0	0	37.8	0	1.0
	>3.5	71.4	82.6	71.4	82.6	4.1
	>4	42.8	95.6	85.7	73.3	9.8
CDR	>0.5	85.7	17.4	38.7	66.6	1.0
	>1	57.1	86.9	71.7	76.9	4.3
	>2	28.6	95.6	80.0	68.7	6.6
	>3	21.4	95.6	75.0	66.6	4.9
MMSE	<17	35.7	91.3	71.4	70.0	4.1
	<20	50.0	82.6	63.6	73.0	2.8
	<23	64.3	65.2	52.9	75.0	1.8

Other factors practically important in the use of each instrument were documented by the administrators and those who participated in the study (Table 2).

**Table 2:** Comparison of selected factors of the study instruments

	MMSE	IQCODE	CDR
Time for administration	About 10 minutes	10–15 minutes	>40 minutes if previous clinical details are not available
Administration of the instruments	By interviewer (some training desirable)	By interviewer	By clinician/trained personnel
Need for informants	Not necessary	Essential	Essential
Limitations of use	Impossible to use in those with hearing/visual disability and illiterate persons	Can be used for all patients provided informants are present	Difficult to use in those with hearing/visual disability
Observation by the researcher	Not wholly culturally acceptable Easy to use	Culturally acceptable Easy to use	Not wholly culturally acceptable Difficult to use

## DISCUSSION

IQCODE was found to be more effective and culturally more acceptable as a screening tool for dementia in Sri Lanka compared with MMSE and CDR.

IQCODE with a sensitivity of 71.4% and a specificity of 82.6% at a cut-off point of 3.5 correlated best with the psychiatric assessment of dementia taken as the gold standard. It also has the advantages of being simple, culturally acceptable, and easy to administer and interpret. IQCODE had a higher specificity than CDR. This is comparable with similar studies where IQCODE was shown to be as good as the MMSE in the diagnosis of dementia.^14^,^15^

In Sri Lanka, although the extended family system is on the wane, reliable informants are still available. This facilitated the use of IQCODE, which is based on informant reports and is more culturally acceptable and reliable. It also has the advantages of easy administration and interpretation when compared to CDR. Nevertheless, informant bias will still be there. Zanetti *et al.*^16^ has showed that the informant's personal characteristics contribute to contrasting results between the informant's reports and direct assessment of activities of daily living in patients affected by mild and very mild dementia. The purpose of administration of IQCODE is to screen for dementia, and those who are found to be positive have to be subjected to detailed clinical evaluation to confirm the diagnosis.

MMSE had the advantages of being quick, easy to use, acceptable to both patients and the assessor without the need for informants to be present. It is impossible to use on patients with hearing impairment or blindness or those who are illiterate. MMSE has been used to screen for dementia in the primary care setting worldwide.^17^ However, in this study, when the cut-off score of 23 was used, the specificity was 65.2% and sensitivity 64.3%. De Silva used a cut-off of 17 yielding a specificity of 90.4% and sensitivity of 94.7%. In our study the corresponding figures were 91.3% and 35.7%, which could probably be due to methodological discrepancies.

Correlation of CDR with the psychiatric assessment showed a specificity of 86.7% and low sensitivity (57.1%) when the standard cut-off of>1 was used. The use of CDR is already a gold standard for the clinical rating of dementia with the advantage of being applicable in a wide range of mild through more severe stages of dementia.^10^ It is time-consuming with the need for detailed knowledge of the individual patient and requires administration by trained personnel. It is mainly used as a clinical rating scale and distinguishes healthy individuals from those with dementia.

In conclusion, IQCODE was found to be the most culturally acceptable instrument, which can be recommended for screening of dementia in the Sri Lankan setting. CDR and MMSE could also be used to screen for dementia in Sri Lanka with due consideration of the advantages and disadvantages of each.
